# Bacterial Community Structure and Dynamic Changes in Different Functional Areas of a Piggery Wastewater Treatment System

**DOI:** 10.3390/microorganisms9102134

**Published:** 2021-10-11

**Authors:** Lin Shi, Naiyuan Liu, Gang Liu, Jun Fang

**Affiliations:** Hunan Provincial Engineering Research Center of Applied Microbial Resources Development for Livestock and Poultry, College of Bioscience and Biotechnology, Hunan Agricultural University, Changsha 410125, China; shilin@stu.hunau.edu.cn (L.S.); liunaiyuan@stu.hunau.edu.cn (N.L.); gangle.liu@gmail.com (G.L.)

**Keywords:** microbial diversity, swine wastewater, activated sludge, wastewater quality

## Abstract

Chemicals of emerging concern (CEC) in pig farm breeding wastewater, such as antibiotics, will soon pose a serious threat to public health. It is therefore essential to consider improving the treatment efficiency of piggery wastewater in terms of microorganisms. In order to optimize the overall piggery wastewater treatment system from the perspective of the bacterial community structure and its response to environmental factors, five samples were randomly taken from each area of a piggery’s wastewater treatment system using a random sampling method. The bacterial communities’ composition and their correlation with wastewater quality were then analyzed using Illumina MiSeq high-throughput sequencing. The results showed that the bacterial community composition of each treatment unit was similar. However, differences in abundance were significant, and the bacterial community structure gradually changed with the process. *Proteobacteria* showed more adaptability to an anaerobic environment than *Firmicutes*, and the abundance of *Tissierella* in anaerobic zones was low. The abundance of *Clostridial* (39.02%) and *Bacteroides* (20.6%) in the inlet was significantly higher than it was in the aerobic zone and the anoxic zone (*p* < 0.05). *Rhodocyclaceae* is a key functional microbial group in a wastewater treatment system, and it is a dominant microbial group in activated sludge. Redundancy analysis (RDA) showed that chemical oxygen demand (COD) had the greatest impact on bacterial community structure. Total phosphorus (TP), total nitrogen (TN), PH and COD contents were significantly negatively correlated with *Sphingobacteriia*, *Betaproteobacteria* and *Gammaproteobacteria*, and significantly positively correlated with *Bacteroidia* and *Clostridia*. These results offer basic data and theoretical support for optimizing livestock wastewater treatment systems using bacterial community structures.

## 1. Introduction

In recent years, with the rapid development of the livestock and poultry breeding industry in China, the problem of pollution emissions has become increasingly serious. Wastewater from livestock and poultry breeding is not only rich in conventional pollutants like chemical oxygen demand (COD), total nitrogen (TN), ammonia nitrogen (NH_4_^+^-N), and total phosphorus (TP), it also contains a large number of chemicals of emerging concern (CEC), as well as antibiotics and various pathogens, all of which pose a serious threat to both public health and ecological security [1].

Biological treatment technology is widely used for livestock wastewater due to the degradation of antibiotics by some resistant bacteria as well as the relatively low cost. At present, the most commonly used biological treatments are the aerobic method, the anaerobic method, and the aerobic–anaerobic mixed method. In aerobic biological treatment, sequencing batch-activated sludge (SBR) is used to treat livestock and poultry wastewater; this process is commonly chosen because of its simplicity, effectiveness in removing COD, NH_3_-N, and phosphorus, good sludge sedimentation performance, and strong adaptability to water quality and quantity changes [2]. The anaerobic method of treating livestock and poultry wastewater uses an upflow anaerobic sludge blanket (UASB) reactor, which has the advantages of high efficiency, low operation costs, and low engineering costs [3]. However, neither anaerobic nor aerobic treatment alone can successfully treat wastewater to the emission standard. Therefore, at present, most domestic large-scale breeding farms utilize anoxic/oxic (A/O) mixed treatment technology to treat their wastewater [4]. The A/O process offers strong resistance to hydraulic shock and adapts well to high amounts of COD. It has a good removal effect on chemical oxygen demand and NH_3_-N, and the effluent quality can meet the discharge standard.

The composition of the microorganism community in the wastewater biological treatment system is diverse, and its level of biodiversity is high [5]. The treatment effect, stable operation and effluent safety of the system are closely related to the microbial community’s structure and function [6,7]. Liu et al. showed that specific microbial communities can promote the degradation of some complex macromolecular organic compounds in wastewater treatment systems [8] while Gu et al. analyzed the microbial communities in livestock and poultry wastewater under four different C: N ratios. Their results showed that the diversity of bacteria and archaea was high in high C: N wastewater, and the removal efficiency of total nitrogen and total phosphorus was the highest [9]. However, most of the current research on microbial communities in livestock and poultry wastewater treatment systems is concentrated on a specific functional area; there are few studies on the structural and dynamic changes in bacterial communities in different functional areas across the entire system. Therefore, the analysis of bacterial communities’ composition in each part of the livestock wastewater treatment system, the identification of a dominant bacterial community, and the finding of a correlation between that bacterial community and pollutant concentrations all play an important role in optimizing the bacterial community structure of the wastewater treatment system and improving water purification efficiency [10].

The traditional bacterial culture method has been widely used in previous research. However, it has been reported that most microorganisms in nature are unculturable bacteria, which makes the bacterial culture method neither comprehensive nor accurate in terms of the study of microbial populations. Moreover, it is difficult to identify the dominant species and the differences between each unit in the processing system using the traditional separation culture method [11]. In recent years, with the development of high-throughput sequencing and analysis technology, domestic and foreign scholars have made steady progress in the study of the microbial communities in soil, intestinal microorganisms, extreme environments, and other complex media [12,13,14,15]. At present, high-throughput sequencing technology is also widely used in the study of the dynamic changes in microbial communities in various wastewater treatment systems and the correlations between microbial communities and various environmental factors [16]; for example, Yan et al. used Illumina MiSeq high-throughput sequencing technology to study the composition of and diversity in the microbial communities in a cattle farm wastewater treatment plant. Their results showed that the dominant phyla throughout the treatment process were *Proteobacteria*, *Bacteroidetes,* and *Firmicutes* [17]. Zhang et al. used Illumina MiSeq high-throughput sequencing technology to study the microbial community structure of the urban sewage treatment system in Chuzhou City and found that *Proteobacteria*, *Chloroflexi*, *Actinobacteria*, *Acidobacteria*, *Actinobacteria*, *Bacteroidetes*, and *Firmicutes* were the dominant phyla [18]. Ospina-Betancourth et al., using Illumina MiSeq 16S rRNA gene amplicon sequencing, defined *Methyloversatilis sp.* as the most abundant species in the reactor for treating high C: N paper mill wastewater [19]. However, there are few reports on the bacterial community composition of the pig farm wastewater treatment system.

With the rapid development of the livestock and poultry industries, the problem of pollution emission is becoming more and more serious. If the wastewater produced during the process is not properly treated, the chemicals of emerging concern (CEC) used in pig breeding, including various antibiotics, will pose a serious threat to public health. Despite this, few of the current studies on wastewater treatment have optimized the piggery wastewater treatment system from the perspective of its bacterial community structures and their correlation with various environmental factors. Therefore, in this paper, the bacterial community structure of a Hunan piggery’s wastewater treatment system was analyzed using high-throughput sequencing technology; the dominant bacteria in different treatment units throughout the piggery’s wastewater treatment system and their dynamic changes during the treatment process were then studied. Additionally, the functional bacteria with degradation in wastewater treatment process were identified and the correlation between environmental factors and changes in the bacterial community was analyzed in order to provide basic data and theoretical support for the optimization of piggery wastewater treatment systems.

## 2. Materials and Methods

### 2.1. Description of Pig Farm and Sample Collection

**Figure 1:**
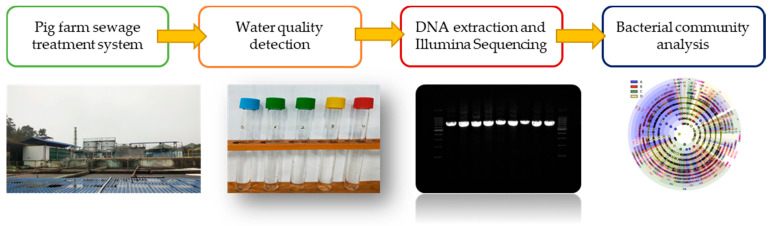
Schematic diagram of experimental process.

The activated sludge samples were taken from Xinguang’an Agriculture and Animal Husbandry Co., Ltd. (Shanghai, China). The water quality of the samples was tested, and DNA was also extracted from them. The bacterial communities in the samples were analyzed using Illumina MiSeq high-throughput sequencing technology (Figure 1). The base wastewater treatment system for pig breeding is in the Changsha, Hunan Province. There are about 400,000 pigs in Xinguang’an and a fecal output of 120 t/d. The piggery wastewater was treated by an anaerobic–anoxic–aerobic biological treatment process. According to the operation of the processing system, a systematic sample collection was carried out on 19 March 2021. The sampling points included an inlet (A), an aerobic zone (B), an anoxic zone (C) and an anaerobic zone (D). Five samples of 500 mL each were randomly collected from each point; 20 samples were collected overall. The collected samples were placed in sterile enzyme-free polyethylene bottles and immediately transported to the laboratory in an ice box and then stored in the laboratory at −20 °C. Sewage quality detection was completed within 48 h post-centrifugation.

### 2.2. Water Quality Detection

In order to investigate the influence of conventional water quality indices on bacterial community distribution, the parameters of conventional water quality, including pH, total nitrogen (TN), total phosphorus (TP), chemical oxygen demand (COD), biochemical oxygen demand (BOD), NH_4_^+^-N, NO^−^_3_-N, and NO^−^_2_-N levels, were determined for each sample. The pH value was determined using the glass electrode method, and the TN level was determined using UV spectrophotometry with alkaline potassium persulfate digestion. The TP level was determined using ammonium molybdate spectrophotometry and COD was determined using the dichromate method. BOD was determined using the dilution and inoculation method. The NH_4_^+^-N level was determined using NaCl reagent spectrophotometry and NO^−^_3_-N and NO^−^_2_-N levels were determined using ultraviolet spectrophotometry [20]. The degree to which a pollutant was removed was found by determining the difference between a given pollutant’s concentration in the previous treatment unit and the current one.

### 2.3. DNA Extraction, PCR Amplification and Illumina Sequencing

The MIO-BIO Power Soil DNA Isolation Kit was used for genomic DNA extraction, and the extraction process was conducted according to the operation manual. The concentration and mass of the extracted DNA samples were found using a Qubit 2.0 Fluorometer (Invitrogen, Carlsbad, CA, USA). DNA samples that met the experimental requirements were sent to Microbiological Technology Co., Ltd. (Shanghai, China). The V3~V4 region of 16S rRNA gene was amplified using primers 357F (5′-ACTCCTACGGRAGGCAGCAG-3′) and 806R (5′-GGACTACHVGGGTWTCTAAT-3′). The PCR was performed in a 50 μL system containing 10 μL of 5xBuffer, 1 μL of 10 mM dNTP, 1 U of Phusion ultra-fidelity DNA polymerase, 1 μL of F/R inner primers (10 uM), and 5 ng–50 ng of template DNA. PCR amplification was conducted using an ABI9700 PCR thermal cycler. The PCR products were recovered via 2% agarose gel electrophoresis. All PCR products were recovered using AxyPrepDNA gel recovery kits and then quantified with an FTC-3000^TM^ real-time PCR instrument. The purified PCR products were then subjected to high-throughput sequencing using the Illumina MiSeq sequencing platform.

### 2.4. Data Analysis

The Illumina MiSeq sequencing data was first spliced using FLASH (version: 1.2.11); the forward and reverse primers in the sequence were then cut using cutadapt (version: 1.16). The low-quality sequences with a Q score of less than 20 were removed for quality control using Prinseq (version: v0.20.4); the chimera was then removed using Usearch (version: 8.1.1861). Finally, the operational taxonomy units (OTUs) were divided according to 97% sequence similarity. The ribosomal database project (RDP) classifier was used to annotate the representative sequences of the OTUs as compared with the database; the confidence threshold default is more than 0.8. The classification information of each sequence from door to species at various levels was then obtained. The statistical analysis of a bacterial community’s structure at various classification levels could then be carried out based on this classification information. The coverage, ACE, Chao1, Shannon, Simpson, and other alpha diversity indices of each biological sample were calculated using Mothur (version: 1.39.5). Excel was used to calculate the number of common and unique OTUs in the four samples while VENN was used to visually show the similarities and overlaps in the numbers of OTUs in the four samples. The weighted UniFrac distance was calculated using QIIME (version: 2 2021.2) for beta diversity evaluation and analysis. The R (version: 3.6.0) language was used for principal coordinate analysis (PCoA) and redundancy analysis (RDA). The differences found in microbial communities from different samples were observed using the PCoA method while the RDA method was used to define the relationship between flora and environmental factors.

All the data in the experiment are expressed as mean ± standard deviation (SD) and analyzed by one-way ANOVA and Tukey’s multiple comparison test to compare the differences between the four groups (SPSS 21 software). *p* < 0.05 was regarded as a significant difference.

## 3. Results and Discussion

### 3.1. Detection of Wastewater Quality

As presented in Table 1, the initial pH value of the piggery’s wastewater was weak alkaline (7.83). The nitrification reaction led to the rapid degradation of ammonia nitrogen and the production of a large amount of H ^+^, which reduced the pH value and wastewater quality and gradually led to weak acidity. The concentration of organic matter in the piggery’s wastewater was high, and the COD concentration in the influent reached levels as high as 3670 mg/L. After anaerobic treatment, the COD concentration decreased sharply to 970 mg/L, and the removal rate reached 73.57%. Wang et al. analyzed the bacterial population characteristics of an industrial petrochemical wastewater treatment plant using a biological treatment process in Liaoning Province, China, and found that the COD removal rate in the wastewater was 81.12% [21]. Although the treatment plant we observed and the one analyzed by Wang et al. processed sewage from different sources, both utilized biological treatment processes. The decrease in COD content in both examples, then, can be seen as the result of the decomposition, metabolization, and digestion of organic matter in wastewater by anaerobic microorganisms. TN and NH_4_^+^-N were minimally affected in the anaerobic zone but largely removed in the anoxic and aerobic zones, with removal rates of 71.23% and 64.38%, respectively. The concentration of NO_3_^−^-N in the aerobic zone was seen to be 60% higher than that in the anoxic zone; this may be due to the advantages of autotrophic nitrifying bacteria under aerobic conditions, as they can stably and efficiently convert NH_4_^+^-N to NO_3_^−^-N [22,23]. At the same time, the occurrence of short-cut nitrification made NO_2_^−^-N (1.48 mg/L) levels much lower than NO_3_^−^-N (33 mg/L) levels in the aerobic zone. TP in the inlet was at 75.2 mg/L; this then decreased to 53.9 mg/L after anaerobic zone treatment, indicating that the phosphorus utilization rate was high at this stage. Ji et al. developed a novel simultaneous nitrogen and phosphorus removal (SNPR) process for the treatment of mainstream wastewater; after 200 days of operation, the removal rates of TN and TP were 93.9% and 94.2%, respectively [24]. Huang et al. created a single-stage biofilm process coupled with anammox and intracellular carbon metabolism (SAIC) for the treatment of swine wastewater after simulated digestion; the removal rate of TN in their SAIC system was 12.77% higher than it was in the reference system, and the removal rate of TP was as high as 83.7% [25]. These results are much higher than the efficiency of nitrogen and phosphorus removal in piggery wastewater. Therefore, the original treatment process of the pig farm can be changed to improve the removal rate of nitrogen and phosphorus in the pig farm wastewater treatment system. The concentration of BOD_5_ in the inlet was 968 mg/L; this decreased sharply after entering the treatment system. In the sewage treatment system, functional bacteria gradually occupied the dominant position and non-functional bacteria were gradually eliminated as the treatment process proceeded, resulting in the decrease in BOD_5_ concentration.

Overall, the piggery’s treatment system was effective in treating the COD, BOD_5_ and NH_4_^+^-N in its wastewater with treatment efficiencies of 83.9%, 73.7%, and 71.2%, respectively. However, its treatment efficiency for TP was only 28.3%.

### 3.2. Diversity Analysis for Bacterial Communities

**Figure 2:**
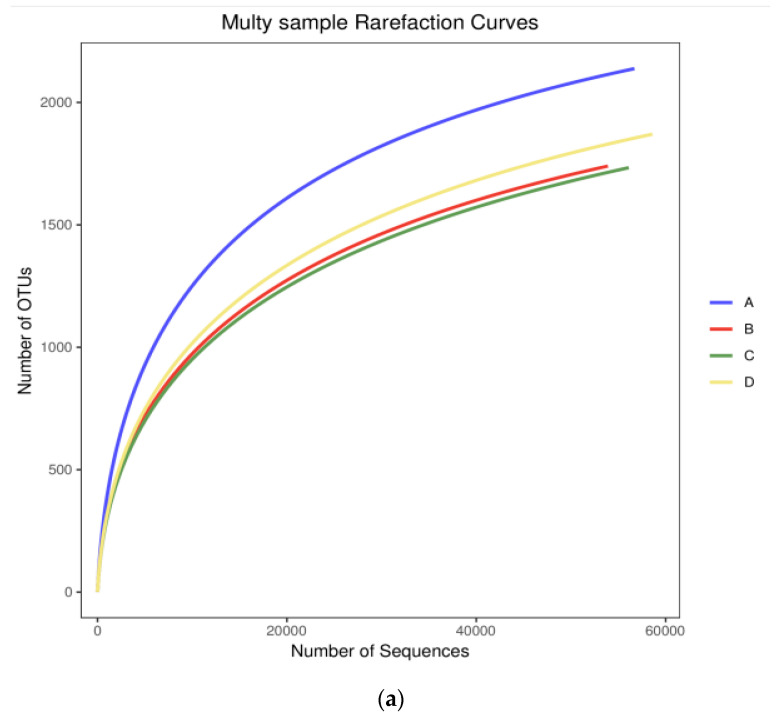

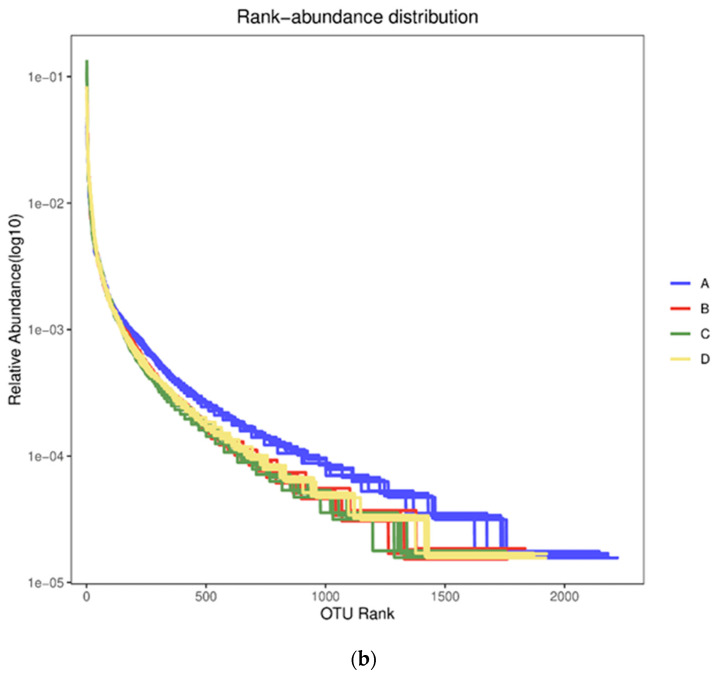
Diversity of bacterial communities in activated sludge samples: (**a**) rarefaction curves and (**b**) rank abundance curves. Note: A: inlet; B: aerobic zone; C: anoxic zone; D: anaerobic zone.

As shown in Figure 2, the rarefaction curve tends to plateau, indicating that our sequencing effort was sufficient for spanning the overall community diversity. Sparse curves in the samples show the high microbial diversity in the original wastewater (Figure 2a). The rank abundance curves of these samples then gradually flatten (Figure 2b), indicating that there are many diverse microbial communities in the different processing units. Among the four processing units, the curves for the aerobic and anaerobic pools decreased the fastest, indicating that the species abundance distribution uniformity in these pools was low. This indicates that there may be dominant strains in the aerobic and anaerobic tanks that are inhibiting the growth of other microorganisms. As shown in Table 2, the microbial community diversity of different treatment units in the piggery’s wastewater treatment system was analyzed using Illumina MiSeq high-throughput sequencing technology. After removing some chimeras produced during PCR and low-quality sequences obtained via sequencing, 238,601 optimized sequences were obtained from the samples; each sample had at least 58,550 valid sequences. OTU coverage showed that more than 97% of microorganisms were captured, indicating that the data obtained in this study sufficiently reflect the bacterial diversity of the specific samples.

The ACE and Chaol indices show the richness of a bacterial community while the Shannon and Simpson indices show its diversity. Alpha diversity analysis results showed (Table 2) that there were differences in the richness index (ACE and Chaol) and diversity index (Shannon and Simpson) between microbial communities in different treatment units. The number of bacterial species and the community diversity in the upstream treatment unit were higher than in the downstream treatment unit (*p* < 0.05). Additionally, the numbers of bacterial species and community diversity in the anoxic zone were higher than those in the aerobic treatment unit. As shown in Table 1, the removal efficiency of TN in the anoxic zone was higher than that in aerobic zone. Zhao et al. found that the system showed higher microbial diversity when the municipal wastewater treatment system achieved deep phosphorus removal and complete nitrification; this shows that the more bacterial species and community diversity exist in the system, the more effective it is at removing pollutants [26]. Yan et al. found that the Ace and Chao indices for a cattle farm’s wastewater treatment system were highest under anoxic conditions, indicating that the community richness of the system increased under those conditions [17]; this was consistent with our results. Most denitrifying bacteria are more suitable for growth under anoxic conditions. This means that the number of bacteria in the anoxic zone will increase due to the proliferation of denitrifying bacteria.

**Figure 3:**
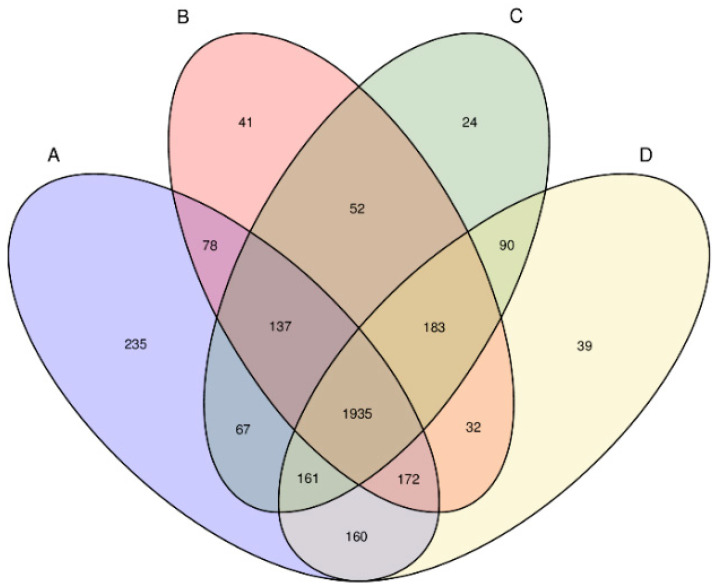
Overlap of the bacterial communities from four sludge samples based on OTU (3% distance). Note: A: inlet; B: aerobic zone; C: anoxic zone; D: anaerobic zone. Note: A: raw wastewater; B: aerobic pool; C: anaerobic pool; D: anaerobic fermenter.

Through a Venn diagram (Figure 3), the similarities and differences of the bacterial communities in activated sludge samples from different treatment units in the piggery’s wastewater treatment system were analyzed using OTU composition. The total number of OTUs shared by the four processing units was 1935, accounting for 56.8% of the total number of OTUs observed (3406). The observation results showed that the same microbial species existed in different treatment pools throughout the piggery’s wastewater treatment system. In addition, there were 235, 41, 24, and 39 unique OTUs in the inlet, aerobic zone, anoxic zone, and anaerobic zone sludge samples, respectively. The unique OTUs accounted for 0.7–6.9%, with an average of 2.2%. The presence of the same OTU in four different treatment pools indicates that the same bacteria exist at different stages of treatment. Ma et al. investigated the microbial communities in three tannery wastewater treatment plants using Illumina MiSeq sequencing; the same microorganisms were found in all three plants, proving that the wastewater source did not directly cause the microbial community structure in the wastewater treatment system [27]. The microbial community structures formed in different processing units differ because of variations in the environmental conditions surrounding them. However, due to the existence of reflux systems in the wastewater treatment process, microorganisms circulate in each treatment unit, meaning that the same microorganisms can be found in different processing units.

### 3.3. Bacterial Community Structure among the Biological Treatment Systems

**Figure 4:**
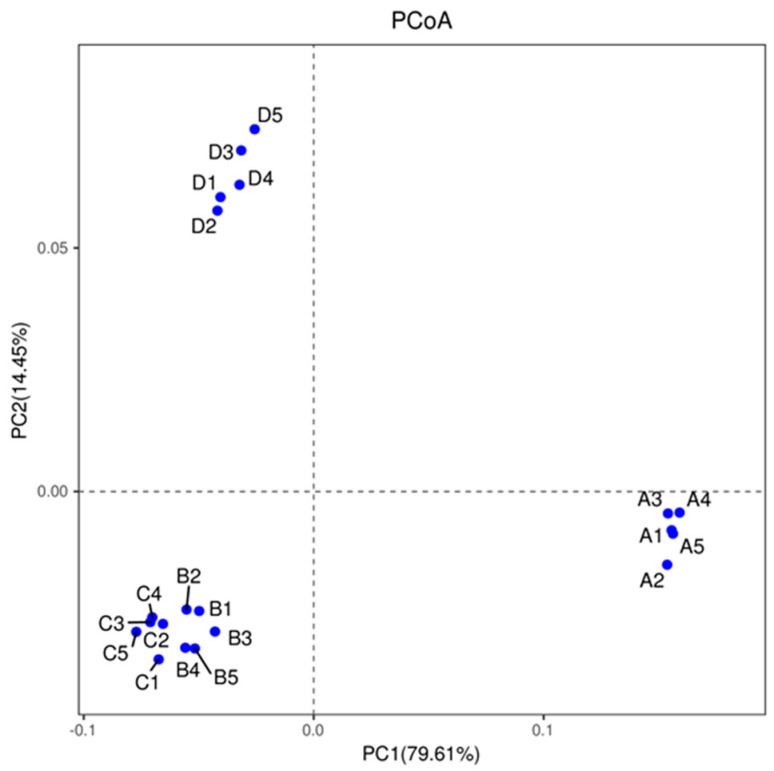
Comparison of the bacterial community structure in each treatment unit of the piggery’s wastewater purification system. Note: A: inlet; B: aerobic zone; C: anoxic zone; D: anaerobic zone.

Principal coordinate analysis (PCoA) is a non-binding data dimension reduction analysis method that can be used to study the similarities or dissimilarities in two sample community’s compositions. As shown in Figure 4, PC1 was 79.61% and PC2 was 14.45%. When using PCoA, the closer the distance between samples, the more similar the community compositions are. The investigation results showed that the microbial community compositions of the aerobic and anoxic zones were similar while the microbial community compositions of the inlet and anaerobic zone had a certain uniqueness. This indicated that the bacterial community composition gradually changed after the wastewater flowed from the inlet zone into the anaerobic zone. Yan et al.’s results regarding bacterial community composition analysis of wastewater treatment plants in cattle farms, therefore, are consistent with ours [17]. As the process progresses, however, the nitrification liquid produced by nitrification in the aerobic zone will return to the anoxic zone while denitrification is carried out in the anoxic zone to achieve nitrogen removal. During the reflux process, some microorganisms in the aerobic zone enter the anoxic zone alongside the reflux liquid, resulting in similar microbial community composition in the aerobic and anoxic zones [28].

**Figure 5:**
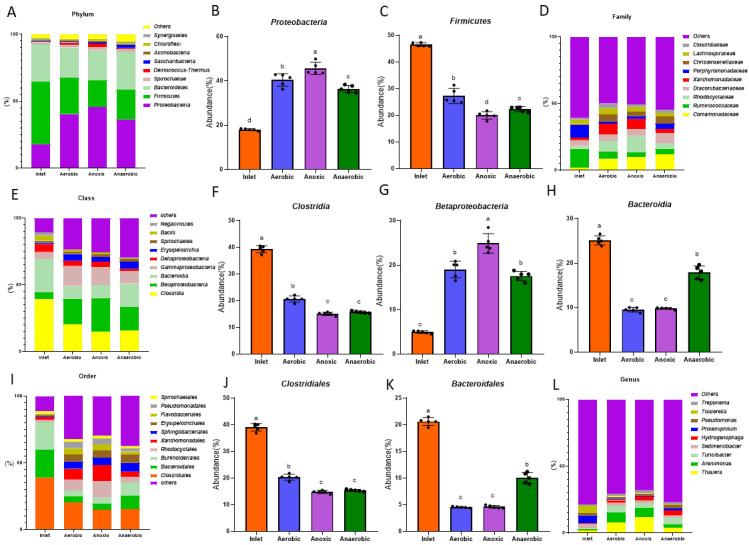
Relative abundance of the microbial community. Note: (**A**) Relative abundance of microbial phyla; (**B**) percentage of *Proteobacteria* in each sample from the four groups; (**C**) percentage of *Firmicutes* in each sample from the four groups; (**D**) relative abundance of microbial family; (**E**) relative abundance of microbial class; (**F**) percentage of *Clostridia* in each sample from the four groups; (**G**) percentage of *Betaproteobacteria* in each sample from the four groups; (**H**) percentage of *Bacteroidia* in each sample from the four groups; (**I**) relative abundance of microbial order; (**J**) percentage of *Clostridiales* in each sample from the four groups; (**K**) percentage of *Bacteroidales* in each sample from the four groups; (**L**) relative abundance of microbial genes. a, b, c, d without a common letter marked indicate significant differences (*p* < 0.05).

An RDP classifier was used to annotate the representative sequences of OTU at a 97% similarity level; 15 phyla-level bacteria as well as some unclassified bacteria were obtained. We selected the ten most abundant phyla for analysis, as shown in Figure 5A, in our study of the wastewater treatment systems in pig farms, we found that the dominant bacteria in activated sludge were *Proteobacteria*, *Chlorolipid*, *Bacteroidetes*, *Spirochetes,* and *Firmicutes*, which is consistent with previous studies. These dominant phyla are quite common in wastewater biological treatment processes; they are dominant bacteria in terms of the degradation effect and therefore participate in the degradation of organic matter in wastewater. The proportion of *Proteobacteria* in the aerobic and anoxic zones was 40.39% and 45.6%, respectively. Kumar et al. found that *Firmicutes*, *Bacteroidetes*, and *Proteobacteria* were the dominant bacteria in swine manure [29]; these were also found to be the dominant bacteria in the piggery’s wastewater, indicating that the bacteria there primarily comes from the pigs’ intestines. With the anaerobic reaction, bacteria suitable for an anaerobic environment began to accumulate in the anaerobic zone. In the anaerobic zone, the species of the dominant bacteria did not change, but the number of bacteria changed significantly. Zhang et al. studied the microbial community structure of activated sludge in the urban sewage treatment system of Chuzhou City using Illumina MiSeq high-throughput sequencing technology and found that the dominant bacteria in their activated sludge samples were *Proteobacteria*, *Acidobacteria*, *Firmicutes*, *Bacteroidetes*, *Chlorolipid*, and *Actinobacteria* [18]. Conversely, when we studied the bacterial communities in the piggery’s wastewater treatment system, *Acidobacteria* was not found to be part of the dominant phyla. The reason for this may be that different sources of wastewater lead to different dominant bacteria in the treatment system. Xu et al. and Luo et al. found that *Proteobacteria*, *Bacteroidetes*, *Chloroliid*, and *Actinobacteria* were the dominant bacteria in the sewage treatment system in Xinjiang and all played an important role in the sewage treatment process [30,31]. Furthermore, Chao et al. found that the dominant bacteria in drinking water were *Firmicutes*, *Bacteroidetes*, *Chlorolipids*, *Actinobacteria*, *Cyanobacteria,* and *Proteobacteria* [32]. As shown in Figure 5B,C, *Proteobacteria* counts (36.37%) in the anaerobic zone were significantly higher (*p* < 0.05) than those in the inlet (17.99%) while *Firmicutes* counts (22.31%) in the anaerobic zone were significantly lower (*p* < 0.05) than those in the inlet (46.51%), which indicated that, at the phylum level, *Proteobacteria* was more adaptable to an anaerobic environment than *Firmicutes*. *Proteobacteria* is one of the most diverse and abundant microbial communities on Earth and it dominates the microbial community composition of activated sludge [33]. Wang et al. used high-throughput sequencing to define the abundance and diversity of bacteria in tannery wastewater treatment plants and found *Proteobacteria* to be abundant in the aerobic zone [34]. The difference between Wang et al.’s results and those in the present study was due to the different sources of sewage, which resulted in different enrichment zones of *Proteobacteria* in different wastewater treatment plants. Wang et al. found that *Firmicutes* were most present under aerobic conditions in the study of microbial communities in urban solid waste landfills [35] while in this study, the *Firmicutes* content in the aerobic zone was higher than it was in the anaerobic zone, indicating that the increase in oxygen is beneficial for their growth.

At the class level, *Clostridia*, *Betaproteobacteria,* and *Bacteroidia* were dominant in the treatment system (Figure 5E). In the anaerobic zone, the levels of *Clostridia* (15.74%) and *Bacteroidia* (17.87%) decreased significantly, while the level of *Betaproteobacteria* (17.53%) increased significantly (Figure 5F–H). Dev et al. found that *Betaproteobacteria* shows strong biodegradability in coking wastewater [36]. Therefore, *Betaproteobacteria* in *Proteobacteria*, as the dominant bacteria in the piggery’s wastewater treatment system, had a stronger removal effect on the organic matter and nutrients in wastewater [37]. Therefore, an increase in the *Betaproteobacteria* level in the aerobic and anaerobic ponds can improve the treatment efficiency of the system. In addition, *Bacilli* was detected throughout the processing system; *Bacilli* is a bacterial class that degrades organic matter in wastewater by producing multiple enzymes. *Bacilli* widely exists in the environment and has the function of degrading antibiotics. It exists throughout the natural environment and degrades antibiotics. *Bacteroidia* lives in the intestines of humans and animals; it can sometimes become a pathogen that threatens human health. Therefore, the elimination of *Bacteroidia* in wastewater treatment systems also has important ecological significance. In the aerobic pool, *Spirochaetales* (1.86%), *Pseudomonadales* (4.81%), *Flavobacteriales* (4.81%), *Burkholderiales* (4.55%), and *Bacteroidales* (4.55%) levels were lower while *Clostridiales* (20.34%), *Rhodocyclales* (8.1%), *Xanthomonadales* (8.1%), *Sphingobacteriales* (5.33%), and *Erysipelotrichales* (5.33%) levels were higher (Figure 5I). As shown in Figure 5J,K, the levels of *Clostridial* (39.02%) and *Bacteroidales* (20.6%) in the inlet were significantly higher than those in the aerobic zone and anoxic zone (*p* < 0.05). Microbial species in the anaerobic tank were like those in the aerobic tank, though their levels differed slightly. For example, *Clostridiales* levels (14.80%) were lower in the anaerobic pool than in the aerobic pool, but *Rhodocyclales* (12.12%) and *Xanthomonadales* (12.12%) levels were higher in the anaerobic pool than in the aerobic pool (*p* < 0.05). The levels of *Bacteroidales* and *Burkholderiales* in the anaerobic fermentation tank were significantly higher than those in aerobic and anaerobic tanks. Although the microbial species in the different treatment pools were similar, their levels varied greatly in each community structure. This difference may be due to the different microbial environments in each treatment pool. Tang et al. studied bacterial community composition in wastewater treatment plants and found that *Clostridiales* were potential bacterial pathogens [38]. *Clostridiales* is a kind of *Clostridia*, which is common in animal feces. After a series of treatments of piggery wastewater, *Clostridiales* can still be detected in the system; at present, China has not strictly defined the emission indicators for pathogenic bacteria in water treatment, which means that the pathogenic bacteria in aquaculture wastewater could then be discharged into the natural environment and threaten human health.

As shown in Figure 5D, the microbial community structure at the family level was significantly different in the inlet than it was in the aerobic, anoxic, and anaerobic zones. The microbial community structure in the anaerobic zone was also slightly different from the microbial community structure in the aerobic and anoxic zones. The dominant families in the inlet were *Ruminococcaceae* (14.32%) and *Porphyromonadaceae* (9.5%). The dominant families in the aerobic zone were *Comamonadaceae* (8.62%), *Rhodocyclaceae* (8.1%), and *Xanthomonadaceae* (8.02%). The dominant families in aerobic zone were similar, namely *Comamonadaceae* (9.77%), *Rhodocyclaceae* (12.12%), and *Xanthomonadaceae* (7.68%). *Rhodocyclaceae* is a key functional microbial group in a wastewater treatment system and is a dominant microbial group in activated sludge. Révész et al. found that *Comamonadaceae* has a strong ability to degrade aromatic hydrocarbons [39] and can play a role in their degradation under aerobic conditions. The increase in *Comamonadaceae* levels in the aerobic zone of the piggery’s wastewater treatment system observed in this study confirmed this conclusion. Wang et al. and Xin et al. used metagenomic studies to show that *Rhodophyta* plays a leading role in denitrification in wastewater treatment systems [40,41]; different genera of *Rhodocyclaceae* have special treatment functions. For example, the studies of McIlroy et al. and Li et al. showed that *Rhodocyclaceae* appears alternately in the enhanced biological phosphorus removal (EBPR) bioreactor under anaerobic and aerobic conditions to enhance the phosphorus removal effect [42,43].

Figure 5L shows the ten most abundant genera. *Proteiniphilum* (5.71%) and *Tissierella* (5.49%) were significantly higher in the inlet than the other three treatment tanks. The level of *Thauera* (11.95%) in the anoxic zone was relatively high while the level of *Tissierella* (0.28%) in the anaerobic zone was low. As the dominant flora in activated sludge, *Thauera* plays a key role in the degradation of phenolic compounds. Semedo et al. found that *Thauera* can not only degrade phenolic pollutants, but also works well in nitrogen and phosphorus removal and denitrification [44,45,46]. However, there are still many deficiencies in the research on the degradation mechanisms of *Thauera*’s aromatic compounds [47]. Tourova et al. found that *Arenimonas* was found to degrade styrene in industrial wastewater [48]. Therefore, the different levels of the dominant genera in the different treatment pools may be related to the pools’ different functions.

Based on the above studies, it was found that the dominant bacteria in the different piggery wastewater treatment stages were not exactly the same. Nitrification is usually carried out in the aerobic zone while denitrification is carried out in the anoxic zone; therefore, the difference in dominant bacteria content in different areas may be related to the different functions of the treatment pools. At the same time, although the treatment system has a certain reduction effect on pathogenic bacteria in livestock wastewater, these bacteria cannot be completely removed or reduced to a relatively negligible level. Several pathogenic bacteria can still be detected in the system, and their subsequent potential impact on the ecological environment and public health cannot be ignored.

### 3.4. Environmental Factor Analysis

**Figure 6:**
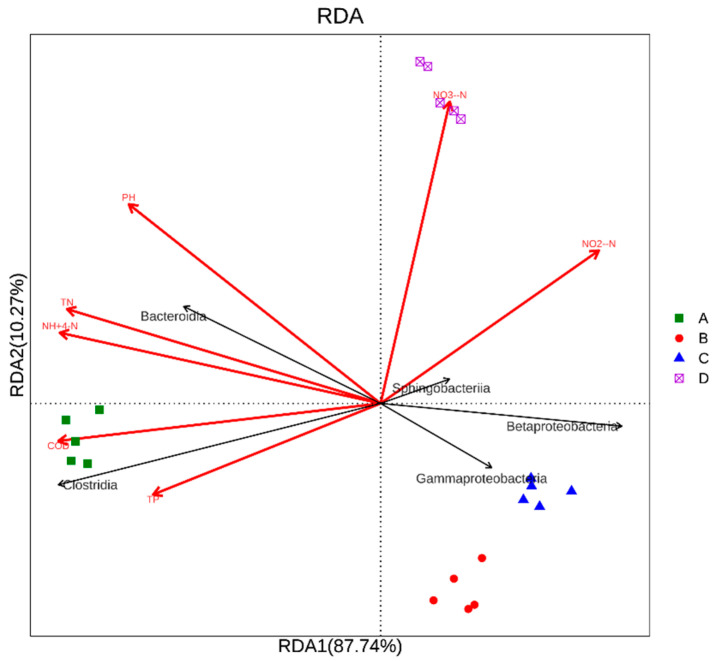
RDA analysis investigating the relationship between microbial communities and environmental variables. Note: A: inlet; B: aerobic zone; C: anoxic zone; D: anaerobic zone.

Water-based environmental factors affect the living environment of microorganisms, and their growth and metabolism, in turn, affect the morphology and concentration of chemical substances in water [49]. RDA analysis, combined with analysis of the water quality in different treatment pools, was performed on bacteria and water environmental factors at the class level. The results, which reflect the relationship between flora and environmental factors, are shown in Figure 6. The RDA analysis showed that the interpretation weights of the first and second axes were 87.74% and 10.27% of the total variance of environmental factors, respectively. The seven environmental variables chosen for study were pH, COD, NH_4_^+^-N, TP, TN, NO_3_^−^-N, and NO_2_^−^-N. RDA showed that the inlet was positively correlated with COD, TP, TN, NH_4_^+^-N, and PH levels and was most affected by COD. He et al. studied microbial communities in five wastewater treatment plants in Beijing and found that TN had the greatest impact on bacterial species [50]. This differs from the results of our study, which showed that COD is the most important factor affecting the composition of microbial communities in piggery wastewater treatment systems. The anaerobic zone was mainly affected by NO_3_^−^-N and NO_2_^−^-N while the aerobic and anoxic zones were negatively correlated with water quality. In addition, *Gammaproteobacteria* was mainly concentrated in the anoxic zone, and negatively correlated with the physicochemical properties of wastewater, especially TN and NH_4_^+^-N. This indicated that *Gammaproteobacteria* had a denitrification reaction in the anoxic zone and that the removal effect of TN and NH_4_^+^-N was obvious. As shown in Figure 5E, the *Gammaproteobacteria* level in the aerobic zone is too high (15%) and contains many pathogenic bacteria. If not completely removed or reduced to a relatively negligible level, its subsequent impact on the ecological environment and public health could be significant. The pH level was significantly positively correlated with *Bacteroidia* and significantly negatively correlated with *Betaproteobacteria*, *Gammaproteobacteria*, and *Sphingobacteriia*. The more *Bacteroidia*, the greater the pH value. *Betaproteobacteria*, *Gammaproteobacteria*, and *Sphingobacteriia* showed the opposite trend. We can therefore adjust the pH value of water by tweaking the interaction between these bacteria. *Clostridia* was significantly negatively correlated with NO_3_^−^-N and NO_2_^−^-N and was significantly positively correlated with TP. Meerbergen et al., in their study of the microbial community composition of textile wastewater treatment plants, found that *Clostridia* are often detected in the activated sludge system, which is composed of a variety of bacteria that decompose pollutants and therefore play an important role in sewage treatment; it has a particularly strong removal effect on phosphorus [51]. Song et al. studied microbial communities in tropical and temperate wastewater treatment plants and found that, if pH, TP, TN, and temperature were different in different treatment ponds, the microbial community structure would be affected [52]. Our analysis results indicated that different external environmental factors have a significant impact on the microbial community composition of the piggery’s wastewater purification system. Therefore, in the treatment of pig farm wastewater, appropriate conditions can be created to inhibit the growth of pathogenic bacteria, promote the proliferation of functional bacteria, and maximize the degradation of organic matter, especially the degradation of antibiotics in water and denitrification, in order to successfully purify the water [53].

## 4. Conclusions

The piggery’s wastewater treatment system had strong treatment effects on the COD, BOD_5_, and NH_4_^+^-N in its wastewater; treatment efficiencies were 83.9%, 73.7%, and 71.2%, respectively. However, the treatment efficiency of TP was only 28.3%. Illumina MiSeq high-throughput sequencing technology was used to analyze the microbial community structure and diversity in different stages of a piggery’s wastewater. The analysis of bacterial community structures showed that the bacterial community composition gradually changed throughout the treatment process. At the phylum level, *Proteobacteria* were more adaptable to an anaerobic environment than *Firmicutes.* At the class level, *Clostridia*, *Betaproteobacteria*, and *Bacteroidia* were dominant in the treatment system. At the order level, the levels of *Clostridial* (39.02%) and *Bacteroides* (20.6%) in the inlet were significantly higher than those in the aerobic and anoxic zones (*p* < 0.05). *Rhodocyclaceae* was found to be a key functional microbial group in wastewater treatment systems and a dominant microbial group in activated sludge. The content of *Tissierella* (0.28%) in the anaerobic zone was low. RDA results showed that the bacterial community structure was most affected by COD while the pH level was significantly positively correlated with *Bacteroidia* and significantly negatively correlated with *Betaproteobacteria*, *Gammaproteobacteria*, and *Sphingobacteriia*. In terms of the bacterial community structure and its response to environmental factors, it was found that the removal rate of TP could be improved by increasing the *Clostridia* content in a piggery’s wastewater treatment system. In this study, the bacterial community in a piggery’s wastewater treatment system was analyzed in depth; scientific data and a theoretical basis were provided for improving the efficiency of the treatment of wastewater from livestock and poultry breeding from the perspective of the response law of the bacterial community and environmental factors.

## Figures and Tables

**Table 1:** Characteristics of wastewater quality in each unit (mg/L).

Sample	pH	COD/mg/L	BOD_5_	NH_4_^+^-N	TP	TN	NO_3_^−^-N	NO_2_^−^-N
Inlet	7.83 ± 0.03	3670 ± 9.06	968 ± 8.34	365 ± 4.41	75.2 ± 2.70	436 ± 9.52	18.9 ± 1.48	0.15 ± 0.01
Anaerobic	7.42 ± 0.03	970 ± 10.38	266 ± 14.31	200 ± 5.74	53.9 ± 6.62	284 ± 8.07	85.3 ± 1.45	26.4 ± 1.16
Anoxic	6.74 ± 0.03	927 ± 12.57	255 ± 15.64	105 ± 5.65	64.4 ± 1.03	177 ± 6.68	13.2 ± 0.65	33.8 ± 1.01
Aerobic	6.32 ± 0.03	984 ± 4.53	263 ± 5.65	130 ± 5.27	54.2 ± 1.67	190 ± 3.14	33.0 ± 1.79	1.48 ± 0.05

Note: Values are mean ± standard deviation (*n* = 5).

**Table 2:** Richness and diversity indices of microbial communities for sludge samples.

Index	Inlet	Anaerobic	Anoxic	Aerobic
Sequences	59833 ± 2774 a	60799 ± 2530 a	59419 ± 2998 a	58550 ± 4079 a
Sobs	2162 ± 49.9 a	1887.6 ± 35.66 b	1761 ± 71.6 c	1777 ± 34.8 c
Chao1	2509 ± 49.5 a	2275 ± 86.2 b	2201 ± 103.1 b	2177 ± 60.2 b
Ace	2504 ± 52.03 a	2308 ± 77.6 b	2207 ± 109.4 bc	2183 ± 45.2 c
Shannon	5.87 ± 0.07 a	5.37 ± 0.05 b	5.15 ± 0.10 c	5.24 ± 0.06 c
Simpson	0.9 ± 0.1 d	2.1 ± 0.1 b	2.5 ± 0.4 a	1.5 ± 0.1 c
Coverage	99.2 ± 0.04 a	99.2 ± 0.05 a	99.2 ± 0.05 a	99.2 ± 0.09 a

Note: Values are mean ± standard deviation (*n* = 5), different letters in the same line indicate significant difference between treatment units (*p* < 0.05), LSD method.

## Data Availability

The data of this study are available from the correspondence author upon reasonable request.
